# A narrative review of visceral leishmaniasis in Armenia, Azerbaijan, Georgia, Kazakhstan, Kyrgyzstan, Tajikistan, Turkmenistan, Uzbekistan, the Crimean Peninsula and Southern Russia

**DOI:** 10.1186/s13071-015-0925-z

**Published:** 2015-06-16

**Authors:** Margarita V. Strelkova, Evgeny N. Ponirovsky, Evgeny N. Morozov, Ekaterina N. Zhirenkina, Shavkat A. Razakov, Dmitriy A. Kovalenko, Lionel F. Schnur, Gabriele Schönian

**Affiliations:** The Sechenov First Moscow State Medical University, Martsinovsky Institute of Medical Parasitology and Tropical Medicine, Moscow, Russia; The Isaev Research Institute of Medical Parasitology, Samarqand, Uzbekistan; The Hebrew University-Hadassah Medical School, Jerusalem, Israel; The Institute of Microbiology and Hygiene, Charite University Medicine, Hindenburgdamm 30, D-12203 Berlin, Germany

**Keywords:** Visceral leishmaniasis (VL), Southern Caucasus, Central Asia, Northern Caucasus, Crimean Peninsula, Causative agents, Reservoirs, Vectors, Diagnosis, Therapy

## Abstract

There is an extensive body of medical and scientific research literature on visceral leishmaniasis (VL) in the Caucasus, Central Asia, the Crimean Peninsula and the southern part of The Russian Federation that is written in Russian, making it inaccessible to the majority of people who are interested in the leishmaniases in general and VL in particular. This review and summary in English of VL in what was Imperial Russia, which then became the Soviet Union and later a number of different independent states intends to give access to that majority. There are numerous publications in Russian on VL and, mostly, those published in books and the main scientific journals have been included here. The vast geographical area encompassed has been subdivided into four main parts: the southern Caucasus, covering Armenia, Azerbaijan and Georgia; Central Asia, covering Kazakhstan, Kyrgyzstan, Tajikistan, Turkmenistan and Uzbekistan; the Crimean Peninsula and the northern Caucasus, which is part of The Russian Federation. Only rare cases of VL have been recorded in the northern Caucasus and Crimean Peninsula. In the other countries mentioned, human VL has been more intense but epidemics like those associated with *L. donovani* in India and East Africa have not occurred. For most of the countries, there are sections on the distribution, clinical aspects, the causative agent, the reservoirs and the vectors. Serological surveys and research into therapy are also covered. Recent studies on VL in Uzbekistan covered the application of serological, biochemical and molecular biological methods to diagnose human and canine VL, to identify the leishmanial parasites causing them in Uzbekistan and neighbouring Tajikistan and the epidemiology of VL in the Namangan Region of the Pap District, Eastern Uzbekistan. More recently, two studies were carried out in Georgia investigating the prevalence of human and canine VL, and the species composition of phlebotomine sand flies and their rates of infection with what was probably *L. infantum* in Tbilisi, eastern Georgia and Kutaisi, a new focus, in western Georgia. Though published in English, summaries of this information have been included where relevant to update the parts on VL in Uzbekistan and Georgia.

## Introduction

**Fig. 1:**
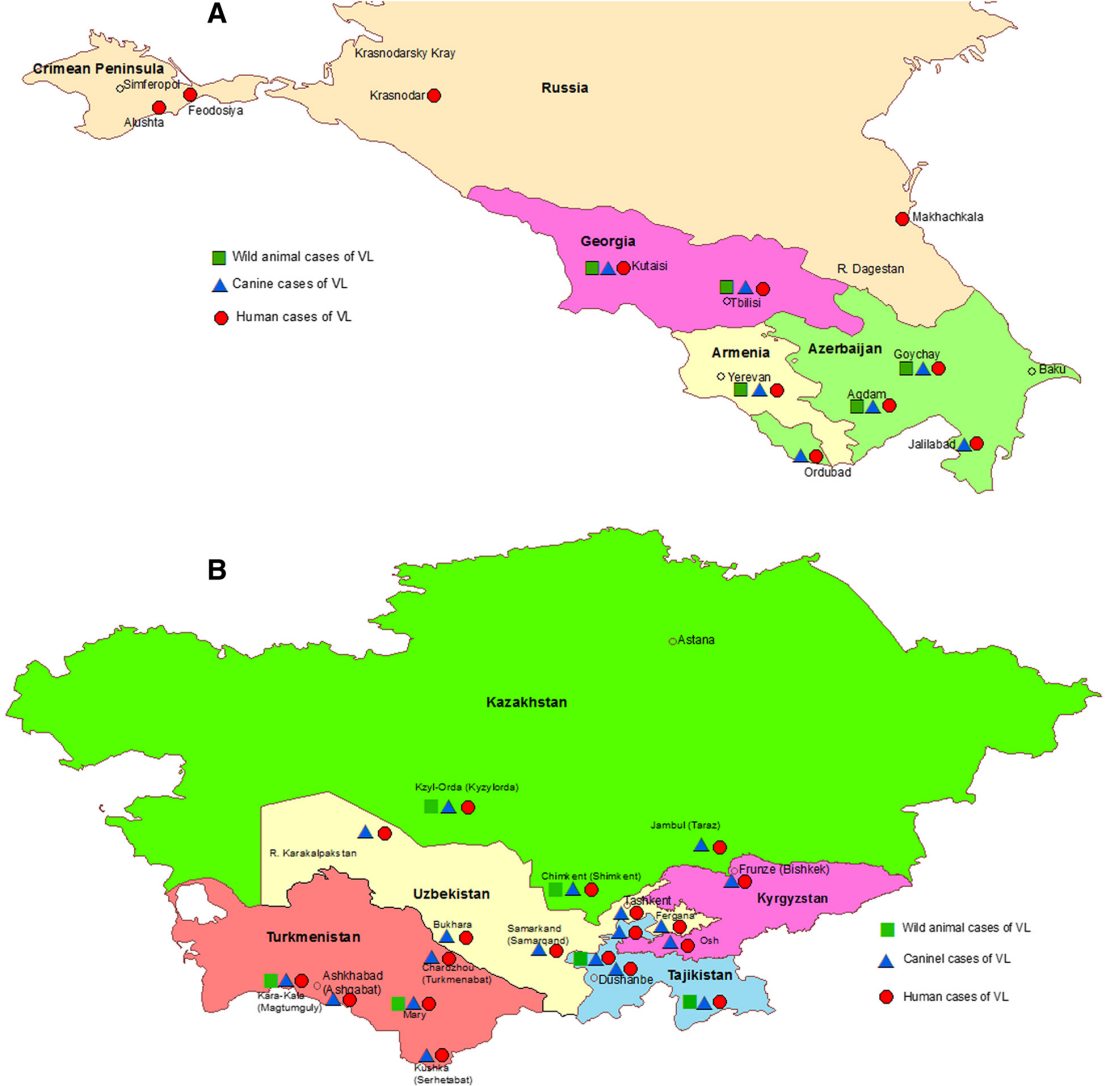
**a**, Distribution of visceral leishmaniasis caused by *Leishmania infantum* in the Crimean Peninsula, the northern Caucasus, the southern part of the Russian Federation, and Georgia, Armenia and Azerbaijan in the southern Caucasus. **b**, Distribution of visceral leishmaniasis caused by *Leishmania infantum* in Kazakhstan, Uzbekistan, Turkmenistan, Tajikistan and Kyrgyzstan in Central Asia

This article reviews visceral leishmaniasis (VL) in: Armenia, Azerbaijan, Georgia, in the southern Caucasus; Kazakhstan, Kyrgyzstan, Tajikistan, Turkmenistan, Uzbekistan, in Central Asia; the Crimean Peninsula; different regions in the northern Caucasus in the southern part of the Russian Federation (Fig. 1a and b), all of which were parts of Imperial Russia, later of the Soviet Union and are now a number of independent states; and is based on articles published between 1911 and 2014. 'Modern leishmaniology', taken here as beginning from the time leishmaniases were first discovered to be caused by protozoal parasites, began in the context of this review during the Imperial Russian period. Visceral leishmaniasis (VL) was well-known to the physicians in Imperial Russia working in the Caucasus and Central Asia from the mid 19th century. In 1915, Yakimov [1] reported on studies done on VL in 1913 during a six-month field trip to Turkestan, which, then, included both Uzbekistan and Turkmenistan of today. Based on his own observations and on a review of the literature of that time, he concluded that VL in that region is identical in humans and dogs and similar to human VL in India and the Mediterranean area. In the time of the USSR*,* VL was studied more thoroughly*,* especially its epidemiology, epizootology and natural distribution and focalization but not to the same degree in different regions. (reviewed in [2, 3]). Following dissolution of the Soviet Union, diseases like VL received less attention, while the now independent republics re-adjusted their medical, public health, and epidemiological services.

Literature pertaining to this subject is very extensive and only those publications that are searchable for the readers have been included here. Most of them are in Russian and this review makes the information presented in them available to readers unfamiliar with Russian. Recent studies on different aspects of VL in Uzbekistan and Georgia were published in English and are also reviewed here for completeness. They covered the sand fly fauna [4, 5], the diagnosis of human cases of VL [6]; and the epidemiology of human and canine VL [7, 8].

Generally, significant numbers of cases of VL were recorded in the southern Caucasus and Central Asia and single ones from the Crimean Peninsula and the northern Caucasus in The Russian Federation over the period covered by this review. The WHO held a meeting in Istanbul in 2009 called the “Consultative Inter-Country Meeting on Leishmaniasis in the WHO European Region”, in which representatives of all the countries mentioned below in this review participated. The numbers of human cases of VL and cutaneous leishmaniasis (CL) recorded for the five years from 2004 to 2008 for the countries represented at the meeting have been listed in the report by Alvar *et al.* [9] together with information regarding treatment and control in the various countries. Table 1 gives the numbers of human cases of VL for that period in the countries considered in this review, which basically indicates a continuation of the trend just describe above. No figures were available for Kyrgystan and Turkmenistan.

**Table 1:** Reported and estimated incidence of visceral leishmaniasis (VL) in the Southern Caucasus, Central Asia, Southern Russia and the Crimean Peninsula for the five years from 2004 to 2008. Data abstracted from Table 5 given in Alvar et al., 2012 [9]

Estimated annual VL incidence	Reported cases/year	Country	Geographical area
10-30	7	Armenia	Southern Caucasus
60-110	28	Azerbaijan	
330-660	164	Georgia	
2-4	1	Kazakhstan	Central Asia
?	0	Kyrgystan	
30-60	15	Tajikistan	
?	0	Turkmenistan	
10-30	7	Uzbekistan	
?	?	Russia	Northern Caucasus
4-7	2	Crimea	Crimean Peninsula

The foci from which cases of VL came were of two types: urban foci where domestic dogs served as the reservoir of the leishmanial parasites and natural foci where wild canids, jackals and foxes, were the putative reservoir hosts [2, 3, 10–12]. Foci involving domestic dogs were urban and until 1960 human cases of VL were almost completely prevented in them by reducing sand fly vector populations during campaigns against mosquitoes that began in the early 1950s to control malaria; and by the elimination of stray and infected dogs [11, 13]. However, foci persisted in rural areas of Georgia, Azerbaijan, Turkmenistan and Kazakhstan where, presumably, transmission included sand fly vectors, wild canids, domestic dogs and people, that enabled the occurrence of sporadic human cases of VL after 1960 with significant fluctuation in the number recorded. A reliable surveillance and control system was developed for such foci that included seasonal observation of sand fly vector abundance and year-round inspection of dogs and the active detection of VL in children. This included the detection of leishmanial parasites in tissue samples. Infected children were hospitalized and treated as part of this surveillance and control. During the existence of the Soviet Union, Solusurmine, a pentavalent antimonial therapeutic, synthesized at The All-Union Research Institute of Chemistry and Pharmaceutics in Moscow, was used to treat human cases of VL. It passed for clinical testing in 1939 [14, 15] and no cases of drug resistance were recorded up to 1979. However, after the dissolution of the Soviet Union the production of Solusurmine stopped, and the acquisition of therapeutics specific for the treatment of human VL cases became problematic in the area under review here.

Identification of causative agents was achieved by several studies carried out for different foci at different times. About 20 stocks of *Leishmania* isolated from people, dogs and a badger were characterized and identified as *Leishmania infantum*, using isoenzyme electrophoretic analysis [16–20]. Several species of *Phlebotomus* belonging to the subgenera *Adlerius* and *Larroussius* were suspected of being the vectors. The vector status of two of these species, *P.* (*A.*) *longiductus and P.* (*L.*) *smirnovi,* was confirmed by a xenodiagnostic method and by comprehensive studies of their ecologies [21–23].

In 2007 and 2008, a consortium of researchers from research institutes in Samarqand, Moscow, Berlin and Jerusalem studied the epidemiology of canine VL in relation to human VL in the Namangan Region of the Pap District in Eastern Uzbekistan. Some of their results have been published, which covered surveying domestic dogs and infant and young children for VL, using clinical signs and symptoms and the application of serological (enzyme-linked immunosorbent assay, ELISA) and molecular biological (internal transcribed spacer 1 region restriction fragment length polymorphism, ITS1 RFLP; multi-locus microsatellite typing, MLMT) methods to diagnose human and canine cases, and biochemical (multi-locus enzyme electrophoresis, MLEE) methods to identify the parasites causing them as being leishmanial parasites and to determine the species of *Leishmania* in Uzbekistan, and in neighbouring Tajikistan [6, 7]. Two recent studies were done in Georgia: one proving *P. kandelakii* and *P. balcanicus* to be vectors of human and canine VL caused by *L. infantum* in Tbilisi [5]; the other exploring the prevalence of human and canine VL and the species composition of phlebotomine sand flies and their rates of infection with what was thought to be *L. infantum* in Tbilisi, eastern Georgia, and in Kutaisi, a new focus, in western Georgia [8].

The situation described briefly above is essentially like that found in many other parts of the world where VL is caused by *L. infantum*. The review moves from country to country within the two main geographical regions (Fig. 1a and b) of this part of the Old World. The locations mentioned in this review are named as they had been in the respective publications; their current names are given in brackets.

## Review

### Methods

This reviews is based on a comprehensive literature search on the distribution, clinical aspects, causatives agents, reservoirs and vectors of VL in Armenia, Azerbaijan, Georgia, Kazakhstan, Kyrgyzstan, Tajikistan, Turkmenistan, Uzbekistan, the Crimean Peninsula and the southern part of the Russian Federation. The retrieved information covers the time period from 1911 to 2014, and came from numerous sources: books; publications in scientific journals; anthologies of research institutions; diploma and doctoral theses; and articles and abstracts of national scientific conferences. Most of these publications are in Russian. This review is mainly based on information published in books and scientific journals that is generally searchable by the readers. In rare cases, data difficult to access were also used. Also included is recent information on VL in the geographical areas covered here published in English in a review by Alvar *et al.* [9] and of some recent articles on studies done on VL in Uzbekistan and Tajikistan [6, 7] and in Georgia [5, 8].

### Southern Caucasus

#### Armenia

##### Distribution

VL was firstly reported in 1913. Between 1935 and 1969*,* 821 cases of VL were recorded from 62 villages in 16 regions of the Republic [24]. The city of Erevan was considered to be the most active focus of VL. A control programme against diseased dogs and sand fly vectors carried out in 1954 and 1955 reduced the morbidity of VL significantly [25]. In 1999, after a long break, a case of VL was diagnosed in a four-year-old child. The number of cases has increased since then with up to 13 cases in 2008 and 10 between January and November in 2009 [9]. In total, 50 cases of VL were registered between 1999 and 2008.

##### Clinical aspects

Most cases were children under the age of 13. However, a 23-year old adult case of VL was reported in 1947 [24].

##### Causative agent

The parasite was not definitely identified to its species but the clinical and epidemiological data suggest it was of the species *L. infantum* [24, 25].

##### Reservoir

In Erevan, infected dogs were the main source of VL in humans [24]. In a search for the wild animal reservoir conducted from 1956 to 1970, 4109 animals (foxes, coypu, wild cats, rodents, reptiles and some domestic animals) were caught and examined [24]. Tissue samples (spleen, liver, bone marrow and blood) from these animals were stained with Giemsa’s stain and examined microscopically. Small amounts of homogenized internal organs were seeded into nutrient NNN culture medium. A culture of promastigotes was isolated from the spleen aspirate of a fox caught near the Village of Jared in the Abovyan Region (now the Kotayk Region) [24]. As foxes are widely spread in Armenia, it was supposed that they were the wild animal reservoir of VL [24].

*Vectors. P. kandelakii* and *P. chinensis* were previously considered to be the primary vectors of the leishmanial parasite causing VL in Armenia [24]. However, according to accepted nomenclature [26, 27] *P. chinensis* is a species found only in China. Sand flies of the *P. chinensis* group were separated into 17 different species, seven of which occurred in what was formerly the USSR: *P. angustus*, *P. balcanicus*, *P. brevis*, *P. halepensis*, *P. longiductus*, *P. rupester* and *P. turanicus*. The Caucasian sand flies previously classified as '*P. chinensis*' are now considered to fall into the three species *P. balcanicus*, *P. brevis* and *P. halepensis.* Of all the species mentioned just above, *P. kandelakii* and *P. balcanicus* are the most likely vectors of VL [28, 29].

Analysis of the distribution of VL in Armenia carried out by Karapetyan and Bagdasaryan (1972) [24] revealed that a temperature not lower than 18 °C for long periods and an average humidity of 45–75 % are required for foci of VL to exist. This was for altitudes between 400 and 700 m.

#### Azerbaijan

##### Distribution

The first case of VL in Azerbaijan was reported in 1913. Between 1913 and 1950, a total of 213 cases of VL were recorded [30] and between 1950 and 1968 1123 (data from the archives of the Institute of Epidemiology, Baku [31]). The cases were unevenly distributed throughout Azerbaijan. Most of them were from the Shirvan Plain in the Geokchai Region (now the Goychay Region), the Kharabah Plain in the eastern foothills of the Lesser Caucasus and the Agdam Region but some were from the mountainous Shamakhi and Ordubad Regions. Owing to the complex measures taken for combating VL that were implemented during an active anti-malarial campaign, morbidity caused by VL in Azerbaijan was reduced, on average, to a twentieth of the original amount by 1966. In the Geokchai, Agdam, Shamakhi and Ordubad regions only a few cases were recorded for eight to ten and even 20 years. In the Jalilabad Region where, previously, only single cases had occurred occasionally, the morbidity began increasing from 1969 and 99 cases of VL were recorded between 1970 and 1979. At the same time, a further 46 cases were reported from all the other endemic regions in the country [31]. A sero-epidemiological survey, employing an indirect immuno-fluorescent antibody test (IFAT), was carried out from 1979 to 1980 on 1580 healthy inhabitants living in five settlements, which revealed that 64 (4.1 %) were sero-positive for VL [32]. From 1984 to 1986, after a break of almost 20 years, 12 cases of VL occurred in the Ordubad Region, nine in the city of Ordubad and three in rural areas. When 3116 healthy people were tested with an IFAT for antibody levels to leishmanial antigens, 170 (5.5 %) of those living in the city and 221 (7.1 %) of those living in nearby villages were found to be sero-positive. This study showed that the percentage of positive reactions was considerably higher for people living in close proximity to cases of VL, 43.2 %, compared to people that lived far away from them, 6.5 % [33]. A total of 347 cases of VL were recorded in Azerbaijan during the 20 years from 1989 to 2009 [9].

In 1987 and 1988, an outbreak of CL caused by *L. infantum* in the Geokchai Region of Azerbaijan was reported with 68 cases being recorded simultaneously in five villages and always with three to five cases in a single family. Twenty (44.4 %) sera of the 45 taken from cases of CL proved positive for anti-leishmanial antibodies, using antigen produced from the Indian strain *L. donovani*, MHOM/IN/1980/DD8. The other 25 (55.6 %) were negative. Serological testing of 62 healthy individuals living in close proximity to the cases of CL revealed that nine (14.5 %) were sero-positive. The highest number of cases was recorded in autumn and winter [20].

##### Clinical aspects

Cases of VL are mainly children under seven years of age and the young age of cases is a typical feature of VL in the Jalilabad Region. Most cases have been newly born infants between five and nine months old. Morbidity decreased sharply among children older than a year and a half. IFATs were negative in children younger than one year old, who had recovered from VL. Only a few of the children between one and a half and seven years of age who had recovered from VL gave positive IFAT result and then with only extremely low levels of antibodies [32]. Cases began occurring in August with most being seen in autumn and winter.

In the outbreak of CL caused by *L. infantum* in the Geokchai Region of Azerbaijan, the ages of the cases ranged, more or less, uniformly, from under one to 50 years old. The cutaneous lesions were localized at the site of the bite of the vector, circumscribed and mainly on the face but sometimes the neck or upper and lower limbs. Many amastigotes were seen in the stained smears made of skin tissue from the margins of lesions. The lesions healed spontaneously and without the need of treatment within one and a half to two years, supposedly, inducing immunity to re-infection with *L. infantum*. While *L.infantum* is generally considered to be one of the causative agents of VL, it has also been shown to cause cutaneous lesions without obvious signs of VL in several countries within the geographical range of the species, *e.g.*, the Pyrenees in France [34, 35], the Abruzzi Region in Italy [36] and in Greece [37].

##### Causative agent

MLEE of eight leishmanial strains isolated from human cases of VL from the Ordubad Region employing a 12-enzyme system indicated they were completely identical among themselves and to a reference strain of *L. infantum,* MCAN/TN/78/LEM78 [33]. Twelve stocks of parasites were isolated from cases from the Geokchai Region, who had VL and also cutaneous lesions. These stocks grew poorly in NNN medium with the number of promastigotes decreasing from passage to passage. Only one stock survived to be characterized by MLEE employing 12 enzyme systems and was identified as a strain of *L. infantum*, i. e., strain MHOM/AZ/1987/Geok-2 (AZ indicates Azerbaijan, its country of origin, which, was previously given as SU for the Soviet Union in scientific articles published before 1991), albeit representing a new zymodeme, on comparing its enzyme profile to those of different Old World reference strains isolated from cases of VL and CL [18, 20].

##### Reservoir

Dogs with VL were found in all the human settlements surveyed. In the Geokchai Region, 470 dogs from six settlements were examined, giving infection rates of 1.4 to 7.1 % [38]. Dogs had chronic infections of VL that lasted a long time and some of the dogs also presented skin lesions in addition to the infection of internal organs like the spleen, liver and bone marrow [30]. In the Ordubad Region, 152 stray dogs were examined between 1985 and 1987, including employing an IFAT. Twenty-seven of these dogs had clinical signs of VL: cachexia, alopecia and hepato-splenomegaly, and 19 (70.4 %) were also positive for anti-leishmanial antibodies by the IFAT. A further 29 asymptomatic dogs were tested and found to be sero-positive [33]. In the Jalilabad Region, five (17.2 %) of 29 dogs investigated were sero-positive by IFAT [32].

A total of 2388 wild animals were examined: cats, wolves, foxes, jackals, porcupines, badgers, hares and different species of rodent and birds [39, 40]. Leishmanial parasites were detected in the internal organs of two foxes and a cat [39].

*Vector. P. kandelakii*, *P. balcanicus*, *P. tobbi*, *P. brevis* and *P. transcausasicus* are considered to transmit VL in Azerbaijan [29, 41]. However, the role of these vector species differs in different regions of the country, depending mainly upon their abundance. For instance, because *P. transcaucasicus* is most abundant in the Jalilabad Region, it is thought to be the principal vector there [42].

#### Georgia

##### Distribution

The first case of VL was recorded in Georgia in 1913. From 1928 to 1999, 1355 local and 15 imported cases of VL were listed in the records of The Virsaladze Institute of Medical Parasitology and Tropical Medicine, Tbilisi. These came from six towns and 164 villages in 19 Georgian administrative sectors, most of which were in the Shida Kartly and Kakheti Regions in eastern Georgia [3]. In the 1970s, cases of VL occurred mainly in the Marneuli and Bolnisi Regions in the eastern part of the Republic bordering Azerbaijan. The number of cases was small, 2–10 per year, sometimes with breaks of 1–3 years. However, in recent years, most cases of VL were reported from Tbilisi in eastern Georgia and its vicinity [8, 43], and a new focus of VL was identified in the West Georgian city of Kutaisi [8]. The number of clinical cases of VL has grown steadily during the last two decades varying between 122 and 189 per year. From 1995 to 2010, 1919 cases of VL were recorded, of which 1052 were from Tbilisi (official disease records, National Center for Disease Control (NCDC), Tbilisi, Georgia). Leishmanin skin testing (LST) was performed on human males and females of various ages from Tbilisi and Kutaisi. In Tbilisi, positive skin tests were 100/688 (14.5 %) and in Kutaisi 21/286 (7.3 %), revealing a significant difference in prevalence between Tbilisi and Kutaisi (*P* = 0.0019). Tbilisi is an active focus of VL and its prevalence is very high in humans and dogs (see below). It is not high in Kutaisi, but it seems to be a newly evolving focus [8].

##### Clinical aspects

Cases of VL are usually children under five years old and, rarely, older children and adults [3]. Georgian physicians used Solusurmine, a preparation of pentavalent antimony produced in the USSR, to treat VL, the daily dose of which was gradually increased with the full daily dose being given only on days 4–5 [44, 45]. This helped to avoid deterioration of the patients’ general condition, especially children below one year old, owing to intoxication caused by the simultaneous killing of the parasites exposed to the therapeutic drug [45]. Treatment was repeated to prevent relapses. This scheme and the well-organised care of patients avoided deaths.

##### Causative agent

Leishmanial strains isolated from humans and dogs were antigenically identical by serotyping and were equally very virulent after injection into Syrian hamsters [46]. A strain, MMEL/GE/1977/Badg* (GE indicates Georgia, its country of origin, which, was previously given as SU for the Soviet Union in scientific articles published before 1991) isolated from a skin ulcer on a badger, *Meles meles*, was characterized and identified as *L. infantum* by MLEE [16, 17]. However, the role of the badger as a reservoir host in the epidemiology of the parasite remains uncertain. Recently, six strains isolated from sand flies, two of which that were isolated from female *P. kandelakii* and one of which was isolated from a female *P. balcanicus* were characterized by sequence alignment of the 70 kDa heat-shock protein gene and identified as *L. infantum* [5]. Eight leishmanial stocks isolated by Bardzhadze in the Marneuli and Bolnisi Regions in 1987 from children aged one to three years old with VL were characterized and identified as *L. major* by MLEE done at The Martsinovsky Institute of Medical Parasitology and Tropical Medicine in Moscow (Strelkova, unpublished data). This was confirmed by excreted factor (EF) serotyping [47] done at The Hebrew University Hadassah Medical in Jerusalem (Schnur and Strelkova, unpublished data). However, there are no records or history of *L. major* existing in Georgia and *L. major* has never been recorded as having caused VL in humans; except for the case of a young girl who developed a generalized infection of leishmaniasis, i, e., CL and VL, caused by *L. major* after she had received a blood transfusion donated by a person with acquired immunodeficiency syndrome (AIDS), after which she too contracted AIDS and her generalized leishmaniasis [48]. The eight Georgian cases of VL and their leishmanial cultures are either the first record of *L. major* existing in Georgia and, also, of VL being caused by *L. major* or they constitute a laboratory error. Hence, this information should be treated with caution, until there is further evidence of *L. major* existing in Georgia and of its causing VL in humans.

##### Resevoir

Stray and domestic dogs were shown to be part of the natural transmission cycle of VL in different Georgian foci [43]. Recently, Babuadze *et al.* [8] examined the sero-prevalence of anti-leishmanial antibodies in the blood of 1575 stray and pet dogs as well as 77 wild canids, 38 foxes and 39 jackals, from Tbilisi and Kutaisi and its vicinity by using the Kalazar DetectTM rK39 rapid diagnostic test. The percentage of sero-positive pet dogs ranged between 21.5 % and 28.1 % in different districts of Tbilisi, and that of stray dogs was 16.1 % In Kutaisi, 17.3 % of the pet dogs and 8 % of the stray dogs were sero-positive. Of the wild animals screened only 2 (2. 6 %), one fox and one jackal, were sero-positive. This confirms earlier findings. In 1965, 19 foxes from the vicinity of the Village of Shaumiany in the Marneuli District were examined, two of which were shown to have had amastigotes in their livers and spleens [49]. In 1980, four jackals were caught close to the Village of Shaumiany, one of which had amastigotes in its spleen and liver [50].

##### Vectors

In Georgia, the sand fly season begins in early June, peaks in July and August, and declines to none early in September. The sand fly fauna is most diverse in the east of the country, the Tbilisi Region and even further east, where 11 different species of sand fly have been identified [51]. *P. kandelakii* and '*P. chinensis*' were thought to be the vectors of the parasitic agents causing VL. However, according to current taxonomy and nomenclature, *P. chinensis* is a species restricted to China and what was the Caucasian ' *P. chinensis* ' constitutes three separate species: *P. balcanicus*, *P. halepensis* and *P. brevis*, of which the former two are found in Georgia [26, 29]. In addition, *P. neglectu*s, *P. tobbi* and *P. transcaucasicus* might also be involved in the transmission of VL. In Georgia, the upper limit for the distribution for *P. kandelakii* is 1240 m above sea level [52].

From 2006 to 2008, Giorgobiani *et al.* [5] collected 1,266 male and 1,179 female sand flies, which fell into five phlebotomine species. Twelve (1.8 %) of 659 female sand flies examined had infections of parasitic flagellates in their digestive tracts: 10/535(1.9 %) were *P. kandelakii*; 2/40 (5.0 %) *P. balcanicus*. The three strains of *L. infantum* that were isolated from female *P. kandelakii* (2) and *P. balcanicus* (1) were genetically very similar (99.8–99.9 %) to strains of *L. infantum* isolated from human and canine cases of VL from the same focus, showing female sand flies of the species *P. kandelakii* and *P. balcanicus* to be vectors of VL caused by *L. infantum* in the vicinity of Tbilisi. For female *P. kandelakii*, blood meal analysis indicated they prefer feeding on dogs (76 %) but also feed on people, endorsing their role as a vector.

In 1961, Maruashvili [53] reported that the phlebotomine sand fly species composition, rates of infection of the sand flies with flagellate parasites assumed to be leishmanial parasites, supposedly, of the species *L. infantum*, and the putative vectors of human VL were different on each side of the River Mtkvari, which flows through Tbilisi. This was recently corroborated by Babuadze *et al.* [8]. The latter study also found that Tbilisi and Kutaisi displayed quite different sand fly populations. Of the five phlebotomine species caught in Tbilisi, 43 % of the 516 female sand flies were *P. sergenti*, none of which were infected, and 45 % were *P. kandelakii*, of which 5.5 % were infected. *P. balcanicus* (53.5 %) and *P. halepensis* (45.8 %), which were the two least prevalent species in Tbilisi, were most abundant in Kutaisi, and flagellates, supposedly leishmanial parasites, were detected in one female of each species.

### Central Asia

#### Kazakhstan

##### Distribution

In Kazakhstan, VL is endemic to the regions of Kzylorda (now Kyzyl-Orda), Jambul (now Taraz), and Chimkent (now Shymkent). In the latter two regions, most of the human cases of VL have occurred in the cities. In the Kzylorda Region, human cases have been more prevalent among inhabitants of small rural settlements. The Village of Karmakchi at 45°35’ north latitude in the Kzylorda Region marks the northernmost border of the distribution of VL in the Eastern hemisphere. Most cases occurred in January, February and March [54]. From 1927 to 1963, 330 human cases of VL were recorded: 240 from Kzylorda; 63 from Chimkent; 18 from Jambul; and 8 imported cases registered in Alma-Ata and one in Semipalatinsk [55]. From 1963 to 1972, 344 case of VL were recorded: 313 from Kzylorda; 29 from Jambul; and two from Chimkent [55, 56]. The last case of VL was reported in 1976 and came from the City of Jambul. Serological surveys, using an IFAT, were conducted in the City of Kzylorda and its vicinity and in the cities of Jambul and Chimkent in 1975–1976. Of the 599 healthy individuals in the Kzylorda Province, of whom 295 were living in settlements where human cases of VL had been recorded during the previous ten years, 16 (5.4 %) were sero-positive. Of the 483 healthy individuals from the City of Jambul, of whom 241 were living in settlements where human cases of VL had been recorded during the previous ten years, 19 (7.8 %) were sero-positive. Among people living far from where cases of VL were recorded, the percentage sero-positivity was 0.9 % in the Kzylorda Region and 2.8 % in the Jambul Region [57]. In the Kzylorda Region, the locations where human cases of VL occurred were connected to natural foci where wild animals, i. e., foxes and jackals, are the sources of the causative agents of human VL. Such foci are characterized by a 5–8 year cyclic increase and decrease in the number of cases [58]. In Jambul and Chimkent, domestic dogs serve as the animal reservoir hosts of human VL. There, the number of cases per year did not vary significantly and decreased sharply after applying anti-leishmanial measures, i. e., elimination of infected dogs and spraying insecticide in homesteads where human cases of VL were recorded and, also, in neighbouring homesteads [56]. From 1992 to 2006, morbidity of VL was maintained at the low level of 0–3 cases per year. No new cases were reported between 2007 and 2009 [9].

##### Clinical aspects

Most cases have been young children. In the Kzylorda Region, 96 % of the cases are children under three years of age, of which 63 % are less than one year old [54, 59]. Of 32 cases of VL that occurred in the City of Jambul, 15 (47 %) were children up to three years old, 12 (37 %) were three to six years old and 5 (16 %) were six to eight years old [60]. The incubation period varied considerably. One infant had developed the disease after an incubation period of 15–20 days, however, on average incubation was about three and a half months. In infants, the disease is acute with only moderate enlargement of liver and spleen, which often leads to a difficult diagnosis. In children between 2 and 5 years old, the liver is considerably more enlarged than the spleen [54]. Most often, patients arrive in healthcare centres 2 to 4 months after the onset of disease, which frequently progresses with complications and is lethal if therapy leading to healing is not achieved within a short time [59]. In acute cases of VL, leishmanial parasites were observed in bone marrow aspirates and also in peripheral blood [54].

##### Causative agent

The strain MHOM/KZ/1975/Dzha*, (KZ indicates Kazakhstan, its country of origin, which, was previously given as SU for the Soviet Union in scientific articles published before 1991) isolated in the City of Jambul from a patient with VL, was identified as *L. infantum* by MLEE [16, 17]. This strain was successfully cultured and grew well in NNN medium for more than 10 years and was maintained by passaging it through Syrian hamsters.

##### Reservoirs

Infected dogs were found in the Kzylorda Region as mentioned above in the section on distribution where leishmanial parasites were detected in 5 (5 %) out of the 100 asymptomatic dogs investigated [54]. In total, 6–7 % of dogs were found infected in the Jambul and Chimkent Regions [55]. In the Kzylorda Region, leishmanial parasites were detected in the internal organs of foxes and jackals [59], and also of a cat and a badger [61].

*Vectors. P. longiductus* and *P. smirnovi* are the proven vectors of VL in Kazakhstan [23]. This was confirmed experimentally by transmitting the causative agent of VL to hamsters by the bites of infected sand flies of these species [21, 62] and by ecological observation in the field [22, 63]. Both species are found around human habitation and both species actively imbibe human blood. *P. smirnovi* is primarily exophilic but is attracted into houses by light [64, 65]. *P. longiductus* is endophilic [66].

#### Kyrgyzstan

##### Distribution

Information on human VL in Kyrgyzstan is very limited. Between 1939 and 1950, 191 cases of VL were recorded mainly from the south of the country in the town of Osh and its environs. Infantile cases of VL were also recorded in the City of Frunze (now Bishkek) and its vicinity [67]. From 1950 to 1968, 166 cases were registered ranging from between 1–2 and 8–22 cases per year with a peak of 22 cases in 1953. No cases have been reported since 1968 [9].

##### Clinical aspects

No information available.

##### Causative agent

The parasite causing VL in Kyrgyzstan has not been isolated and, therefore, not characterized and identified.

##### Reservoirs

One dog with clinical signs of canine VL was found in the town of Osh [67].

##### Vectors

In the 1960s, *P. longiductus*, (formerly considered to be *P. chinensis,* see above), and *P. caucasicus* were incriminated as the putative vectors of human VL in Kyrgyzstan [68, 69]. However, data collected in other foci where human VL has occurred in what was the USSR has not confirmed the role *P. caucasicus* as a vector of VL.

#### Tajikistan

##### Distribution

Human VL was reported in the cities of Dushanbe, Leninabad (now Khujand) and Ura Tube. From 1929 to 1935, six cases were recorded from the vicinity of Ura Tube. From 1933 to 1934, more than 30 adult and infantile cases were recorded from the settlement of Vakhshstroy [70]. From 1946 to 1958, 33 cases were recorded, 23 of whom were citizens of Dushanbe. The rest were from the surrounding rural districts. Four cases of VL were registered between 1970 and 1979 [56]. In 2002 and 2003, seven Tajik cases of VL from the Aini and Penjikent Regions were seen and treated in the hospital of the Isaev Research Institute of Medical Parasitology in Samarkand [6]. In total, 194 cases were recorded from 1994 to 2008 with between four to 19 cases per year [9]. Fifty VL cases were reported in 2009, mainly from the Penjikent Region. However, it is not clear whether these represent locally acquired infections or imported cases of VL from neighbouring countries [71].

##### Clinical aspects

The very limited information indicates that most cases are young children. A six-month old feeble male patient, with pale skin, little appetite, anaemia, hepato-splenomegaly, a blood count with decreased numbers of eosinophils and leucocytes, and an accelerated erythrocyte sedimentation rate (SER) was treated at the Isaev Institute of Medical Parasitology, Samarkand, with 2 ml of Glucantime per day for 12 days after the case of VL had been microscopically confirmed and then discharged from the hospital in a satisfactory condition (unpublished data), showing the same situation seen in classical cases of VL described for the Mediterranean Region.

##### Causative agent

Alam *et al.* [6] recently identified the parasite causing human VL in Tajikistan while identifying the parasite causing human infantile VL in Uzbekistan. They found that the parasites isolated from two human cases of infantile VL contracted in 2006 in the Penjikent Region of Tajikistan, which neighbours Uzbekistan, were strains of *L. infantum* and very similar to those that had caused cases of VL in Uzbekistan. This was done by extracting and amplifying DNA by PCR and applying PCR-sequencing of the ribosomal ITS1 region [72], and also by MLMT, using 14 microsatellite markers specific and polymorphic for strains of the '*L. donovani* complex' [73, 74]. In this case, the DNA was extracted from the amastigotes in preparations of bone marrow aspirates that had been stained with Giemsa's stain for microscopy.

##### Reservoirs

Infected dogs were shown to be the source of human cases of VL during the period from 1946 to1949 [70, 75] and infected jackals were found in the vicinity of the settlement of Vakhshstroy [70].

##### Vectors

Petrishcheva (1936) [76] and Perfiliev (1966) [77] added the species *P. major* and *P. chinensis*, two species that might serve as vectors of VL, to the sand fly fauna of Tajikistan. However, according to the more recent taxonomic revision of the subgenus *Phlebotomus* (*Adlerius*) by Artemiev [26], *P. major* exists only in India and *P. chinensis* only in China. In 1995, Volkova [78] studied the sand fly fauna in Tajikistan and found three species of the subgenus *Adlerius*: *P. longiductus*, *P. turanicus*, and *P. angustus*, all previously considered to be *P. chinensis*, and two species of the subgenus *Laroussius*: *P. kandelakii* and *P. keshishiani*, both previously considered to be *P. major*.

#### Turkmenistan

##### Distribution

At the beginning of the 20th century, VL was not an obvious and widespread disease in Turkmenistan. One human case of VL was reported from Chardjou (now Turkmenabat) in 1911 [79], a second from Ashkhabad (now Ashgabat) in 1913 [1], a third from Tagtabazar in 1928 [2] and another 193 between 1934 and 1943 [80]. Human cases of VL have come mainly from the cities of Chardjou, Ashkhabad and Mary, the smaller towns, and the adjoining rural areas in the Murghab and Amu Darya Valleys and on the piedmont plains of Kopet Dag. Morbidity increased significantly to 136 cases in the shorter period between 1960 and 1966. During the 15 years from 1966 to 1981, no human cases of VL were reported in Turkmenistan [81]. New ones began appearing and during the three years from 1981 to 1983 54 human cases of VL were recorded [82], and for the whole period between 1981 and 1987 a total of 79 cases were recorded [9]. The vast majority of these cases occurred in the area around Mary in south-eastern Turkmenistan [82, 83]. Sero-epidemiological surveying done in south-eastern Turkmenistan, using an IFAT, revealed 1.6 % sero-positivity among the 681 healthy individuals checked, which included 517 children and 164 adults [84]. In 1988, Lesnikova [85] examined 130 healthy children up to 10 years old, who lived in two settlements in south-eastern Turkmenistan where the risk of infection was high. Of the 23 children that were less than three years old, 11 contracted VL between 1982 and 1987. Sero-epidemiological surveys, again using an IFAT, revealed 24.0 % sero-positivity among children less than three years old in one village and 60.9 % in another village. Extensive investigations of VL and CL in Turkmenistan exposed their distribution, epidemiology and epizootology, and enabled the classification of different foci in a cadastre based on landscape topography, indicating the risk for acquiring VL and CL in Turkmen foci [81]. In addition, Ponirovskii [86] summarized the results of studies done on the epidemiology and epizootology of human VL in Turkmenistan, accentuating the cyclical activation of foci of VL related to heliogeophysical factors.

##### Clinical aspects

The disease mainly affects children up to three years old. The clinical manifestations of the disease are like those seen in Mediterranean infantile VL [80, 82]. Adults very rarely contract the disease and only two adult cases have been recorded since 1985, both of whom were of non-specifically compromised immune status before the onset of the disease.

##### Causative agent

In 1963, three strains were isolated from human cases of VL and grown on NNN rabbit blood-agar slopes. Culture was successful only when parasites were isolated during the early stages of the disease and not later than two months. The isolated parasites grew slowly within the first 15 days but later grew normally. In tissue cultures promastigotes transformed into amastigotes after 24–48 hours. Attempts were made to infect 20 white mice, 16 Syrian hamsters, four hedgehogs, eight cats, three puppies, six foxes and one jackal but succeeded in only one puppy. Three months after being infected, the puppy became feeble, lost its appetite and presented partial alopecia. No leishmanial amastigotes were detected in stained liver and spleen smears however promastigotes were successfully grown from isolated liver tissue incubated in NNN rabbit blood-agar medium [80]. One of the three strains, MHOM/TM/63/VL (TM indicates Turkmenistan, which, was previously given as SU for the Soviet Union in scientific articles published before 1991), was used to prepare lyophilized antigen [32, 87] that was employed successfully in an IFAT for diagnosing VL and in sero-epidemiological surveying [84, 87]. Twelve strains were isolated between 1985 and 1990: eight from human cases of VL, of which seven were from children 1–3 years old and one was from an adult; and four from canine cases of VL [19, 85, 88]. By MLEE, employing eight enzymes (GPI, PGM, 6PGD, MDH, G6PD, ME, ALAT, ASAT), these strains were very similar to a reference strain, MCAN/TN/1978/LEM78, of the species *L. infantum* and, to a lesser extent, to that of the reference strain, MHOM/IN/1980/DD8, of the species *L.donovani*. The strains from the human cases represented a quite homogenous group. They encompassed two different zymodemes that showed a different electrophoretic mobility for only their GPI. Interestingly, the five strains isolated from human cases of VL from the south-eastern part of the Karakum Desert belonged to one of these zymodemes, which was distinct from the zymodeme to which the two strains isolated from human cases of VL from the western Kopet Dag region belonged. Three of the canine strains, of which two were isolated from dogs from the Kopet Dag region and one from a dog from south-eastern part of the Karakum Desert, were characterized by MLEE and differed from the strains from human cases of VL in the electrophoretic mobilities of two of the eight enzymes checked, MDH and ASAT, but the mobility of their GPI was the same as that of the human strains from Kopet Dag and the reference strains of the species *L. infantum* and *L. donovani*. The dog from which the fourth leishmanial strain, MCAN/TM/1990/IOL-5, was isolated came from the south-eastern part of the Karakum Desert. Its leishmanial strain differed from those from the people and the other three dogs, respectively, and also from the reference strain, MCAN/TN/1978/Lem-78, of the species *L. infantum* in its enzyme profile [85]. Thus, the strains of *L. infantum* that had caused human and canine VL in Turkmenistan were genetically highly similar, but not completely identical [19]. Whether this means that canine and human VL is genetically isolated needs to be tested using a higher number of human and canine isolates and modern molecular approaches with higher discriminatory power. Syrian hamsters were infected with nine of the 12 strains, four of which came from the western focus at Kopet Dag and five from the south-eastern focus in the Karakum Desert. Six were from human cases of VL and three were from dogs [85, 88]. The hamsters were injected intra-peritoneally with amastigotes in splenic tissue taken from a previous series of hamsters that had been infected with the strains. On average, clinical signs of leishmaniasis were observed in the test hamsters in 70 days. These included feebleness, loss of appetite, decrease in body mass, dishevelled fur and partial alopecia. At autopsy, the absence of fatty tissue and turgor of the gut was seen. In all cases, the spleen was enlarged, of reddish colour and porous. Generally, the liver was not enlarged. No differences were seen in the clinical manifestation and duration of disease that could be related to strains' geographical origins with regard to their coming from either the western piedmont plains of Kopet Dag or from the south-eastern part of the Karakum Desert, or whether they were isolated from human or canine hosts. The strain MCAN/TM/1990/IOL-5, isolated from a dog and belonging to a distinct zymodeme was not investigated in hamsters. It proved impossible to cultivate the strains from the human cases and dogs in normal NNN rabbit blood-agar medium for more than two passages even using different modifications of the medium's liquid phase. Experimental infection with strains isolated from human cases was successful in just one out of three dogs and in 12 out of 18 cotton rats (*Sygmadon hispidus*) but failed in three wolves, one fox, nine great gerbils (*Rhombomys opimus*), eight Libyan jirds (*Meriones libycus*), 82 white mice, 12 white rats, 28 Natal multimammate mice *(Mastomys natalensis*) and three Nile rats (*Arvicanthus niloticus*) [85].

##### Reservoirs

Yakimov [1] was the first to describe, both, human and canine VL in what was then called Turkestan, and referred to a much larger area than that of Turkmenistan of today. He diagnosed VL with certainty in seven (7 %) dogs from Ashgabat, one (2.5 %) dog from Mary and four (23.5 %) dogs from Kushka (now Serhetabat) among the many dogs he examined by detecting amastigotes in smears of their splenic tissue stained with Giemsa's stain. Dogs with VL were also discovered much more recently in the Murghab Valley [89]. Lesnikova & Sabitov [88] cultured four leishmanial strains isolated from infected dogs, of which two of the dogs came from the western piedmont plains of Kopet Dag and two from south-eastern part of the Karakum Desert (see above). Amastigotes were seen in smears of tissues from internal organs of one fox from south-east Karakum [85], two jackals from Kala-Kala (now Magtumguly) [89] and a porcupine (*Hystrix indica*) from a place between Kala-Kala and Kyzyl-Atrek (now Etrek) [90]. No further identification of these parasites was undertaken. Foxes were suggested to be the natural reservoir hosts in the south-east Karakum region [85] because they concentrate around areas of human settlement and their population increases in some years. If this is so, foxes could act as either a direct source of human infections or an indirect one via dogs.

##### Vectors

In previous publications, *P. chinensis, P. mongolensis* and *P. caucasicus* were considered to be the vectors of the leishmanial parasites causing VL in Turkmenistan [91]. In Turkmenistan, the species *P. turanicus,* previously classified as '*P. chinensis*', is endemic to the Turanian zoo-geographical region [27] and now the one considered to be the vector of VL there [88]. *P. turanicus* is found in rodent burrows, wild animal dens and lairs and places of human habitation on the loess-piedmont plains in the valleys and the piedmont plains of the inhospitable Karakum Desert. Female sand flies of the species *P. turanicus* feed on, both, dogs and people. Significant variations have been seen in the abundance of the species. Human cases were recorded during years when the index of abundance of *P. turanicus* exceeded one sand fly per one standard sheet of paper in oil traps [85].

#### Uzbekistan

##### Distribution

Knowledge of the existence of VL in Uzbekistan dates back to the early part of the 20th century when 30 human cases were recorded from five Uzbek cities: Tashkent, Andijan, Bukhara, Samarkand (now Samarqand), and Termez [92]. During the early and mid-20th century, Uzbekistan, out of all republics in the USSR, recorded the highest number of human cases of VL. Between 1920 and 1985, 6112 human cases of VL were recorded, most of which were from settlements in the Zarafshan and Fergana Valleys and the large cities in Uzbekistan, notably, Kokand, Tashkent and Samarkand [93]. In the Zarafshan Valley, 1503 human cases of VL were recorded between 1935 and 1964, 1064 (70.8 %), most of which came from Samarkand [94]. In the Fergana Valley, human cases of VL were from the cities of Namangan, Andijan and Fergana and the areas around them. In the Namangan Region, 113 human cases of VL were recorded between 1948 and 1953, of which 81 came from the city of Namangan [95]. In the Andijan Region, 142 cases of VL were recorded between 1945 and 1956, 46 of which came from the City of Andijan and 96 from adjacent areas [96]. Large-scale measures for detecting and removing infected dogs, started in Tashkent in 1926 by Khodukin and his colleagues and in Samarkand in 1932 by Isaev and his colleagues, together with anti-mosquito insecticide spraying to curtail malaria that also reduced sand fly abundance around the cities reduced the occurrence of VL to single sporadic cases by the end of the 1960s [97, 98]. Official reports by the Uzbek authorities recorded only 108 cases of VL between 1960 and 1987 [99].

Retrospective screening of the population was carried out in the Provinces of Samarkand, Navoi, Bukhara, and the Kashkadarya Regions, and the Autonomous Republic of Karakalpakiya, which were formerly very active foci of VL. From 1985 to 1988, 8652 people were tested in a sero-epidemiological survey, using an IFAT. Sero-positivity was low on average at 0.38 %, indicating the success of the control measures undertaken [99]. Now the Pap District of the Namangan Region in eastern Uzbekistan has become the main focus of VL in Uzbekistan [4, 6, 7, 100, 101]. The first case in the Pap District was recorded in 1987 [102]. In total, 95 human cases of VL were recorded from nine villages of the Pap District from 1987 to 2009 [7]. In contrast, single human cases of VL have been reported from the Autonomous Republic of Karakalpakiya in western Uzbekistan. In 2007 and 2008, within the framework of an INTAS international project on VL in Uzbekistan, 514 blood samples taken from children under 14 years of age from four villages of the Pap District, Chodak, Oltinkan, Gulistan and Chorkesar, were tested in a sero-epidemiological survey, using an ELISA. Of all those tested, 10 % were sero-positive that included active cases of VL at the time of testing and previously cured cases. Antibodies against the causative agent of VL were detectable in 35 (7.7 %) of 454 healthy children [100]. In another diagnostic study, three bone marrow samples from three active VL cases, nine of venous blood and 222 of peripheral blood were analysed by a PCR-based diagnostic test for detecting leishmanial DNA. The three bone marrow samples were positive as were six (46.2 %) peripheral blood samples from among those from 13 children that had previously had VL, ten (37 %), some venous and some peripheral blood samples from 27 children that were hospitalized owing to other diagnoses and 85 (44.7 %) peripheral blood samples from the 190 healthy individuals tested [101].

##### Clinical aspects

Age composition analysis of human cases of VL showed two out of three were children under the age of three and the incubation period was one to six months but mostly two to three months [103]. Mirzoyan [103] noticed the appearance of a spot or papule, visible by eye, on the tender skin of infant and very young cases of VL, following the bite of an infected sand fly. When the contents of these papules were examined microscopically within one to two months of their appearance, but not later, leishmanial amastigotes could be detected. He called this the “primary effect” as it was the site of entry and initiation of the infection, and the primary focus of inflammation. Half of the patients showed this “primary effect” and after four to eight months leishmanial amastigotes could be detected in stained smears of bone marrow. Cases were admitted to hospitals after a rise in temperature was observed. Mirzoyan also described an efficient, shortened regime for treating VL with Solusurmin [14, 104]. Kassirsky [105] contributed significantly to the clinical diagnosis of VL by urging the use of sternal puncture as being safer than splenic puncture, and advised using a special needle with a limiter for puncturing the sternum. In the study done in 2008 and 2009, most of the human cases of VL from the Pap District, as previously mentioned, were children between the ages of one and three (24, 77.4 %). One case (3.3 %) was a child less than one year old, three (9.7 %) were between four and seven years old, and three (9.7 %) were older than seven years [7, 100]. The disease progresses with loss of appetite and body weight, reduced activity of the children, increasing anaemia and, resulting from this, changes in skin colour and subsequent hepato-splenomegaly. The results of the serological and PCR-based diagnostic surveys in healthy children from the Pap District indicated the possibility of undetected asymptomatic infections of VL [100, 101].

##### Causative agent

Many of the earlier studies addressed the question of VL in humans and dogs being caused by the same agent. Yakimov [1] succeeded in infecting dogs with infected tissues samples from human cases of VL, which was confirmed by others [10, 106], leading them to conclude that the causative agents of human and canine VL are identical. Until quite recently, it proved impossible to isolate the parasites and culture them for characterization and identification. This was overcome by Alam *et al.* [6], who amplified DNA, extracted from the amastigotes in preparations of bone marrow aspirates that had been stained with Giemsa's stain for microscopy, and then applying PCR-sequencing of the ribosomal ITS1 region [72] and MLMT using 14 microsatellite markers specific and polymorphic for strains of the *'L. donovani* complex' [73, 74] to that DNA. One adult human case of VL and ten infantile ones from the Namangan Region and two from the Jizzakh Region of Uzbekistan were categorically shown to be caused by *L. infantum*. These Uzbek strains of *L. infantum* were very similar to strains that had caused two human cases of VL in 2006 in the neighbouring Penjikent Region of Tajikistan, showing them also to be strains of *L. infantum* [6].

Ten stocks of parasites were isolated from lymph node and spleen aspirates taken from dogs and three were isolated from bone marrow aspirates taken from human cases of VL from the area encompassing the villages of Chodak, Oltinkan, Gulistan and Chorkesar in the Namangan Region of the Pap District, Eastern Uzbekistan, during the survey done in 2007 and 2008 [6, 7]. They were cultured and characterized by MLEE of 15 of their enzymes [107, 108] by staff of the Université Montpellier 1, Centre National de reference des *Leishmania*, Laboratoire de Parasitologie-Mycologie, CHU de Montpellier, Montpellier, France. All thirteen stocks were identified as strains of *L. infantum* belonging to zymodeme MON-1, the most ubiquitous zymodeme of this species. That the causative agent of human and canine VL in Uzbekistan is *L, infantum* was corroborated by sequencing the ITS1 amplification products of the ten strains from the dogs and the three from human cases of VL mentioned above. Microsatellite profiles were generated through MLMT for eight of the ten strains from dogs. They had the same genotype by MLMT and, in this, were identical to some and very similar other strains of *L. infantum* that had caused human cases of VL from the study area and the Jizzakh Region [6, 7]. MLMT revealed that the Uzbek strains were a unique and distinct genetic group when compared to strains belonging to the zymodeme MON-1 from other geographical locations [7].

##### Reservoirs

Infected dogs are the main animal reservoir and source of VL in the human population. Based on detecting leishmanial amastigotes in smears of splenic aspirates stained with Giemsa’s stain, 116 (27,2 %) of the dogs examined in Tashkent were found to be infected, as were 20 (40,8 %) of the dogs in Samarkand and 27 (25,2 %) of the dogs in Old Bukhara [1, 106]. Most of the infected stray dogs did not present overt clinical signs of the disease. A few pedigree domestic dogs presented the following symptoms: weight loss, alopecia, swelling of the nose, paralysis of the hind limbs. Khodukin [12] who examined thousands of dogs found, that conjunctivitis and blepharitis of the eyes were cardinal symptoms of canine VL and seen in 95.9 % of infected dogs. The clinical manifestations and pathogenesis of canine VL were studied in detail later by Isaev and Ryabtsev [11] and Isaev [97]. They described skin lesions and other dermal defects on diseased dogs, usually on the head in the mucous membranes of the nose and lips, and also on the ears where amastigotes were found and served as the source of infection of female sand flies. However, leishmanial parasites were never found in the peripheral blood of the thousands of dogs investigated and whose blood smears were examined microscopically. Wild animals have only rarely been studied in Uzbekistan and VL has not been detected among them.

In 2007 and 2008, a study on canine VL was carried out in the villages of Chodak, Oltinkan, Gulistan and Chorkesar and their environs, which are situated in the Namangan Region of the Pap District [7, 100]. Owned dogs from homesteads where human cases of VL had been recorded during the five-year period from 2002 to 2007 and those from homesteads within 300 meters of them, and some stray dogs were surveyed, 162 of which were examined and screened for canine VL. Blood samples from them were assayed for the presence of leishmanial DNA using a PCR-based diagnostic test and sera separated from the blood samples were assayed for antibodies against antigen prepared from leishmanial promastigotes using an ELISA. Lymph node and splenic aspirates were taken from those presenting clinical signs and symptoms of canine VL and seeded into normal rabbit blood-agar semisolid medium, and what later proved to be ten strains of *L. infantum* were isolated. Forty-two dogs (25.9 %) had clinical signs suggestive of canine VL and 51 (31.5 %) were sero-positive for anti-leishmanial antibodies. Of 119 asymptomatic dogs, 24 (20.2 %) were sero-positive. No association was found between the degrees of sero-positivity and either the age or gender of dogs or the particular village of their domicile but a significant one was found between sero-positivity and the presence of suspect clinical signs and symptoms of VL (p < 0.001). ITS1-PCR-sequencing was done on blood and tissue samples from 135 dogs, 40 (29.6 %) of which proved positive for leishmanial infections. Four foxes caught in the general area that were examined and screened similarly to the dogs, were not found to be infected.

*Vectors. P. longiductus* and *P. smirnovi* are the putative vectors of human and canine VL in Uzbekistan, the former being widely dispersed in areas of human settlement and the natural biotopes of the Fergana Valley [4]. The entomologists who proposed this also found that 46 % of the phlebotomine sand flies they caught were *P. sergenti*, 18.8 % *P. papatasi*, 15.5 % *P. longiductus*, 10.3 % *P. alexandri* and 9.6 % were other species. During an epidemiological survey of human infantile and canine VL carried out in the same region in 2007 and 2008, about 10,000 sand flies were caught that showed a very similar species composition to that described by Maroli *et al.* [109]. Of these, *P. longiductus* was considered the most likely to be the vector of *L. infantum* in this region as it was found transmitting the parasite in other parts of Central Asia [4, 23]. In Karakalpakiya, where single human cases of VL have recently been reported, *P. smirnovi* is possibly the vector where it is found on Tugai trees in the floodplains of the Syrdarya and Amudarya rivers.

#### Crimean Peninsula

##### Distribution

Up to 1990, only four confirmed human cases of VL were reported from the Crimean Peninsula. These infections were acquired in the vicinity of Feodosiya and on the coast between Sudak and Alushta [110–112]. From 1990–2007, seven more human cases of VL were reported, most of whom were considered imported, mainly from Tajikistan and Armenia. Another three were diagnosed from 2008 to 2009, two of which were fatal. They were registered as citizens of Kiev and Lvov, who had visited and become infected either in Feodosiya or its vicinity. One case of HIV/*Leishmania* co-infection was diagnosed in 2009 [9].

##### Clinical aspects

In three infant cases of VL, the disease was acute with the typical clinical signs of weight loss, splenomegaly and fever, and proved fatal in two. In contrast, the disease proceeded relatively smoothly in three adult cases but, owing to a late diagnosis, resulted in the death of one. One case was described in detail. The patient, a Lithuanian, acquired his VL in August 1981 during a short tour in East Crimea. In March 1982, after 6-7months, he felt feeble and suffered short bouts of high temperature and sweating. Only in October 1983, did he seek medical advice, was hospitalized and diagnosed with acute chronic bronchitis, and only in January 1984, when his liver, spleen, inguinal and axillary lymph glands became greatly enlarged, was a sternal puncture done and large numbers of leishmanial amastigotes were detected in his bone marrow smears and lymph node aspirates. He was treated with a course of Glucantime and successfully cured [111].

##### Causative agent

Tissue from an inguinal lymph node of the Lithuanian patient mentioned above was inoculated into NNN rabbit blood-agar slopes and also injected intra-peritoneally into ten young Syrian hamsters. Culture was successful and the strain MHOM/CR/1984/Krim (CR indicates Crimea, its country of origin, which, was previously given as SU for the Soviet Union in scientific articles published before 1991) was sub-passaged 35 times before being cryo-preserved. However, growth of the promastigotes was always rather moderate [111]. The strain was characterized by MLEE, using a system of 11 enzymes, and identified as a strain of *L. infantum* [16]. When analysed by molecular biological methods, strain MHOM/CR/1984/Krim always grouped with strains of the species *L. donovani*, but could not be assigned exactly to a specific sub-group and occupied an intermediate position between *L. donovani* and *L. infantum* in phylogenetic trees [74, 113, 114]. By MLMT analysis, its microsatellite profile placed it most closely to strains of *L. donovani* from India and Kenya that belonged to the zymodeme MON-37 and zymodemes close to zymodeme MON-37 [115]. However, the strain MHOM/CR/1984/Krim, itself, does not belong to the zymodeme MON-37 and is listed as belonging to the zymodeme MON-73.

##### Reservoirs

There are no records of studies on possible animal reservoirs of VL in Crimea. Kellina *et al.* [111] suggested foxes as the most likely reservoir hosts for the infectious agent and source of human infection.

##### Vectors

The sand fly fauna of the Crimea was studied intensively during the first half of the 20th century owing to the emergence of sand fly fever there at that time. According to current records and nomenclature, several phlebotomine species were found to exist in Crimea that included *P. longiductus*, a proven vector of VL in other parts of its range, and *P. balcanicus, P. perfiliewi* and *P. neglectus*, which are considered to be putative vectors of VL [27, 116].

### Russia

#### Northern Caucasus

##### Distribution

Stable foci of VL do not appear to exist in any of the Russian territories. However, opinions differ on whether local transmission of the parasites causing human and canine VL could arise in a region like the Northern Caucasus. On comparing natural and climatic conditions, Malkhazova and Neronov [117] concluded that natural transmission is unlikely to occur. However, the presence of sand fly species belonging to the phlebotomine subgenera *Larroussius and Adlerius* known to transmit the causative agents of VL, supposedly *L. infantum*, elsewhere, supports such a notion, should the causative agents somehow be introduced. Only two autochthonous human cases of VL have been reported from the Russian Federation: one, an adult who had always lived in and never left Dagestan [118]; the other, a child from Krasnodar [2]. The first case was reported in 1938; the second in 1923. During the 20th century, human cases of VL tended to be examined and diagnosed in clinics in places in non-endemic areas of the former USSR and were registered as coming from these places. Thus, human cases of VL recorded throughout the years were registered according to their current place of domicile, *e.g.*, Moscow, Mogilyev, Kazan, Irkutsk, Tomsk, Leningrad (now St. Petersburg), though they were actually infected in places where VL was endemic, *e.g.*, Tashkent, Kokand, Ashkhabad, Chardzhoy and Crimea [112]. Fourteen cases of VL, six adults and eight children, where diagnosed among Russian citizens by staff of the Martsinovsky Institute of Medical Parasitology and Tropical Medicine, Moscow, between 1995 and 2011, having acquired their infections in Azerbaijan, Armenia, Georgia, Turkmenistan, Ukraine, Greece and Iran.

##### Clinical aspects

Not described.

##### Causative agent

Not isolated.

##### Reservoirs

Not investigated. The rare occurrence of human cases led to the conclusion that wild animals, especially jackals and foxes, might serve as a source of human infections. Infections of VL have been detected in jackals and foxes in other regions, including ones bordering Russia. The current extension of the distribution of jackals in the northern part of the Main Caucasus Ridge [119] suggests jackals could serve as the reservoir of human and canine VL.

##### Vectors

Owing to the almost non-existence of VL in the North Caucasus, vectors thereof have not been studied and identified; however the sand fly fauna in the North Caucasus was studied extensively during the 1940s and 1950s. The studies were undertaken in regions where cases of sand fly fever had been recorded. Far less information was collected on sand flies during the second half of the 20th century however information and results collected, collated and published previously were put into reviews published between 1989 and 2011 [29, 51, 116]. The City of Armavir, on the 45° north latitude in the Krasnodar Region where *P. neglectus* was caught, was found to be the most northerly point of sand fly distribution in the North Caucasus [120]. *P. balcanicus* and *P. perfiliewi*, putative vectors of human and canine VL, were also found in the Krasnodar Region [120]. *P. balcanicus* and *P. tobbi* were found in the Stavropol Region, and *P. kandelakii, P. balcanicus* and *P. transcaucasicus* in Chechniya [121, 122]. In Dagestan, sand fly distribution is confined to the area between the coastline and the foothills with the City of Kizlyar [120], which included *P. kandelakii* and *P. neglectus*, two putative vectors of human and canine VL.

## Conclusions

This review covers a century and more on the surveillance of and research on human and canine VL in Central Asia, the Caucasus and the southern part of the Russian Federation. Much of it is based on the history of VL during the time of Imperial Russia and the Soviet Union. In the 20th century, the majority of the articles were published in Russian and only sporadically in English, which made it very difficult for the experts in other countries to get information from this vast area. Studies and research like those of Maroli *et al.* [4], Alam *et al.* [6], Kovalenko *et al.* [7, 100], Abdiev [93] and Zhirenkina *et al.* [101] on VL in Uzbekistan, and Giorgobiani *et al.* [5] and Babuadze *et al.* [8] in Georgia have helped to update the situation in Uzbekistan and Georgia but similar studies and research do not seem to have been undertaken elsewhere in the geographical landmass considered here. Surveillance and research should be encouraged and undertaken in the other countries covered in this review.

VL caused by *L. infantum* in the total area is zoonotic and very similar to that of the Mediterranean and Middle Eastern regions, and, though very worthy of consideration, does not appear to be as serious a problem as the anthroponotic VL caused by *L. donovani* which results in epidemics in India, Sudan, Ethiopia and Kenya.

Nevertheless, until the mid-20th century, VL presented a serious public health problem for the countries mentioned in this review because significant numbers of the cases of VL were infants and children, and some form of control was necessary. The national malaria control programme undertaken in the USSR in the 1950s also led to a significant decrease in the sand fly population in addition to the mosquito population. Together with the antimalarial control measures, dogs with VL in areas of human population were eliminated. This reduced human VL to sporadic cases and the disease even ceased to exist for many years in some areas.

However, since 2000, VL has again become a problem for the health authorities in many of the countries mentioned in this review. This is related to the resurgence of locally acquired cases of VL and also to imported cases resulting from increased human migration, travel, *etc.* Also, climate change, for whatever reason, could lead to the extension of the regions of endemicity of VL.

Access to chemotherapeutics for the treatment of VL is problematic in almost all of the independent states that arose after the dissolution of the Soviet Union. Antimonial drugs, *i.e.*, Glucantime, are registered only in Georgia where treatment is financially supported by the government (K. Sidamonidze, pers. communication, WHO meeting Istanbul, 2009). In all the other countries, these chemotherapeutics are not registered, special permission is required for their importation and, thus, the supply is not continuous. Often the chemotherapeutics, mostly antimonial preparations and, rarely, Amphotericin B, have been made available through personal initiatives, *e.g.* by the patients’ relatives, and through humanitarian aid by the WHO. As in other countries with few cases of VL, it is very difficult for medical practitioners to obtain the small quantities of medicine needed [123].

Now, further surveillance and research should be done to expose the existing situation regarding VL in all the countries mentioned in this review. This should include the older well-studied foci where the situation might have changed with time and areas that have the potential of developing into new foci of VL. New leishmanial isolates from human cases, dogs, wild animals and sand flies are required to identify causative agents, animal reservoirs and vectors of VL and for comparison with strains from neighbouring territories, especially where isolates have not been identified, e.g., Armenia; Kyrgyzstan; and in Georgia where, many years ago, eight strains isolated from children with VL were identified as *L. major*, an unexpected result, probably, indicating a laboratory error.

The present situation calls for new approaches to research on VL in the vast area that was considered here, and, in fact, also CL owing to the occurrence of cases of CL caused by *L. infantum* but not displaying obvious signs and symptoms of VL, and the introduction and implementation of modern serological and molecular biological assays for the routine diagnosis of VL. This together with modern methods of identifying isolates will assist in exposing asymptomatic human cases of VL and also choosing treatment for patent human cases of VL. The research done through international joint projects in Uzbekistan and Georgia shows the great benefit of such collaboration for the implementation of modern diagnostic methods and the development of local expertise in the countries mentioned in this review.

## Abbreviations

VL: Visceral Leishmaniasis
CL: Cutaneous Leishmaniasis
ELISA: Enzyme-Linked Immunosorbent Assay
IFAT: Indirect immuno-Fluorescent Antibody Test
ITS1 PCR-RFLP: Internal Transcribed Spacer 1 Region PCR-Restriction Fragment Length Polymorphism
MLMT: Multi-Locus Microsatellite Typing
MLEE: Multi-Locus Enzyme Electrophoresis
LST: Leishmanin Skin Testing
EF: Excreted Factor
AIDS: Acquired Immunodeficiency Syndrome
SER: Erythrocyte Sedimentation Rate
